# Contrat unique, une approche innovante de financement du niveau intermédiaire du système de santé en République Démocratique du Congo : processus et défis de mise en œuvre

**DOI:** 10.4102/phcfm.v13i1.2869

**Published:** 2021-12-06

**Authors:** Ghislain Bisimwa, Samuel L. Makali, Hermes Karemere, Christian Molima, Raphael Nunga, Alain Iyeti, Faustin Chenge

**Affiliations:** 1 École Régionale de Santé Publique, Faculté de Médecine, Université Catholique de Bukavu, Bukavu, République Démocratique du Congo; 2 Centre de Connaissance en Santé en R.D Congo, Kinshasa, République Démocratique du Congo; 3 Hôpital Provincial Général de Référence de Bukavu, Bukavu, République Démocratique du Congo; 4 Direction d’Études et de Planification, Ministère de la Santé Publique, Kinshasa, République Démocratique du Congo; 5 École de Santé Publique, Faculté de Médecine, Université de Lubumbashi, Lubumbashi, République Démocratique du Congo

**Keywords:** Financing, health system, basket fund, intermediate level, DRC

## Abstract

**Basket fund, an innovative approach for intermediate health system level financing in the Democratic Republic of Congo: Implementation process and challenges**.

**Background:**

Universal health coverage should allow countries to establish a financing strategy in order to guarantee the health of the population.

**Aim:**

Our objective was to describe the process and preliminary results of the implementation of the basket fund approach as a mode of financing the intermediate level (provincial health divisions) of the Congolese health system.

**Setting:**

The study was conducted in the provincial health divisions (PHDs), representing the intermediate level of the health system in the Democratic Republic of Congo, where the basket fund approach has been implemented

**Methods:**

We conducted a mixed-methods convergent study as part of the evaluation of the basket fund approach in the Democratic Republic of Congo, five years after its introduction (2014–2019). Data was collected through a document review and individual interviews by telephone. A descriptive analysis of the quantitative data was conducted using Statistical Package for Social Sciences (SPSS) version 24 software. The qualitative data were analysed by thematic analysis using a pre-established thematic framework.

**Results:**

The implementation of the basket fund approach was effective in some (PHDs) (53.8% in 2016). The operating costs of the PHDs varied according to the size, density and number of health zones covered. In the PHDs where the basket fund was operational, this approach appeared to contribute to improved planning and management in the use of resources, the partnership between technical and financial partners (TFPs and PHDs) and incentives for the performance of PHD agents.

**Conclusion:**

In the DRC, the basket fund approach has contributed to improved collaboration between donors in the health sector and facilitated the decentralisation of funding planning to the provincial level.

## Introduction

La couverture universelle en santé (CUS) est l’une des cibles du troisième Objectif de Développement durable (ODD3)^1^. Pour réussir la mise en œuvre de la CUS, chaque pays devrait mettre en place une stratégie de financement garantissant l’accès aux soins à toute sa population tout en réduisant au maximum les paiements directs. L’Organisation Mondiale de Santé (OMS) encourage les pays à faible et moyen revenus (PBMR), y compris les « États fragiles » à mettre en place des réformes pour améliorer les performances de leurs systèmes de santé^2,3^. Ces réformes sont généralement focalisées sur la sauvegarde des six piliers du système de santé tels que définis par l’OMS : prestations des services, ressources humaines, financement, information sanitaire, médicaments et équipement ainsi que gouvernance et leadership^4^.

La mobilisation, la coordination et la gestion efficiente du financement de la santé constituent un axe essentiel dans la mise en œuvre de la CUS. Cependant dans les PBMR, le financement de la santé repose essentiellement sur l’aide au développement et une participation importante des ménages aux coûts des soins^5,6^. La coordination de sources variées de financement est le principal défi évoqué par les chercheurs intéressés par la problématique du financement de la santé dans les pays en crises et dans les états fragiles^7^. Une étude récente sur les réformes des systèmes de santé de cinq pays d’Afrique subsaharienne ayant connu des conflits armés (Angola, Erythrée, Ethiopie, Mozambique et Rwanda) montre que la décentralisation des systèmes de santé, le renforcement des agents de santé communautaires et la réforme du financement de la santé des gouvernements sont les réformes qui ont contribué à la réduction de la mortalité maternelle (reflet de la qualité des soins) d’au moins 50% dans ces pays^5,8^.

En République Démocratique du Congo (RDC), la mortalité maternelle est estimée à 692 décès maternels pour 100000 Naissances Vivantes (NV)^8,9^ et reste parmi les plus élevées au monde. La mortalité infanto-juvénile quant à elle s’est nettement améliorée au cours des deux dernières décennies, allant de 205 décès pour 1000 NV en 2001^10^ à 70 décès pour mille NV en 2018^11^.

Concernant le financement du secteur de la santé, une enquête réalisée par le Ministère de la santé (MSP) en 2014^12^ avait montré une disparité dans la répartition des Partenaires Techniques et Financiers (PTF) et dans l’allocation des financements au niveau des provinces. Certaines provinces avaient plus de 15 PTF alors que d’autres n’en avaient que deux ou trois. Aussi, plus de 55% du financement alloué aux provinces était orienté vers les programmes spécialisés et seulement 18% de ce financement étaient orientés vers l’appui des plans d’action opérationnelles de la Division Provinciale de la Santé (DPS). L’équipe cadre de la DPS était débordée par le nombre de contrats signés selon les projets et les PTF. Par exemple une DPS avait signé jusqu’à 33 contrats pour bénéficier des financements de PTF différents^12^. Les provinces n’étaient pas impliquées dans les négociations des financements parce que tout était planifié à partir de Kinshasa, souvent sur base d’un canevas de planification standard pour toutes les provinces sans tenir compte des spécificités de chacune.

Pour répondre aux problèmes du système de santé, le MSP et ses partenaires avaient amorcé le processus de réforme en période post-conflit, en élaborant la Stratégie de Renforcement du Système de Santé (SRSS) en 2006, révisée en 2010^13,14^ qui prend en compte les différents piliers du système de santé. En ce qui concerne le pilier « financement », la réforme prônée par la SRSS consiste en une nouvelle approche visant à décentraliser la coordination et les lieux de négociation des financements vers les provinces en vue de mettre en place des « basket fund » provinciaux par des contrats uniques (CU) et éviter la rétention des financements au niveau central par les structures de coordination nationales, jugées trop bureaucratiques et coûteuses^14^.

Il s’agissait, dans le cadre de cette réforme, de mettre en œuvre une approche visant à mettre en commun les différentes ressources destinées à soutenir les activités des DPS (financements de partenaires, financement du gouvernement et autres sources potentielles des financement). Ce processus a commencé en 2014 dans un contexte de réforme du niveau intermédiaire et se poursuit jusqu’à ce jour^15^.

Cet article vise d’abord à décrire le processus de mise en œuvre du CU comme mode de financement du niveau intermédiaire du système de santé en RDC et, ensuite à présenter les résultats préliminaires ainsi que les défis observés dans les provinces qui ont démarré la mise en œuvre de cette politique de réforme stratégique du financement de la santé au niveau intermédiaire du système de santé.

## Encadré 1 : Définition du concept « contrat unique »

C’est une approche qui consiste à mettre ensemble toutes les ressources disponibles de manière transparente quelle que soit l’origine (État, PTF) pour soutenir de manière globale l’atteinte des missions des DPS. Le contrat a pour objet la mise en commun des appuis financiers au sein de la DPS en tant que structure chargée de la coordination, de l’encadrement et de l’accompagnement technique des zones de santé (ZS) en vue d’améliorer la qualité de l’offre des soins et les conditions sanitaires de la population de la province.

Le CU représente ainsi le cadre au sein duquel sont rassemblés tous les appuis financiers alloués par le gouvernement et les partenaires techniques et financiers du secteur santé pour appuyer la mise en œuvre du plan d’action opérationnel de la DPS. Cette approche a été adoptée par le MSP lors de sa réunion de comité national de pilotage du système de santé (CNP-SS)^15,16^.

## Méthodologie

### Cadre d’étude

La RDC est un pays d’Afrique Centrale. Sa population est estimée à 90 millions d’habitants^13,14^. Elle est, par sa superficie de 2 345 000 km^2^, le deuxième pays le plus vaste d’Afrique après l’Algérie. Le pays est divisé en 26 provinces administratives. Les voies de communications sont déficitaires (réseau routier moins praticable, quelques lignes aériennes entre certaines villes et à un rythme irrégulier) rendant ainsi les contacts entre l’administration centrale et les provinces difficiles^17^. C’est un pays classé dans la catégorie des « États fragiles » du fait de son instabilité depuis près de 3 décennies avec des longues périodes des conflits armées^18,19,20,21^. Depuis des décennies, la proportion du budget de l’État alloué à la santé varie entre 4 et 6%^22,23^. Plus de 70% du financement de la santé proviennent de l’aide au développement^22,23^.

Le système de santé de la RDC compte trois niveaux. Le niveau central (composé du Ministre de la santé et son cabinet, du Secrétariat général à la santé, de l’Inspection générale de la santé, des directions centrales et des programmes spécialisés) qui élabore les normes et donne les grandes orientations en matière de politique de santé. Le niveau intermédiaire est composé de 26 DPS et inspections provinciales de la santé (IPS) correspondant aux 26 provinces administratives. La DPS a pour mission principale d’assurer l’encadrement de proximité des ZS. Le niveau opérationnel est constitué de ZS, au nombre de 516 au total, qui assurent la mise en œuvre des soins de santé primaires (SSP) à la population^24^.

Conformément à la Constitution du pays^25^, la mise en œuvre des SSP en RDC est décentralisée et revient à la compétence des provinces et les DPS jouissent d’une autonomie relative de fonctionnement. Les subsides pour le fonctionnement proviennent généralement des PTF. Les interventions de l’État sont essentiellement limitées à couvrir les salaires du personnel et certaines interventions de réhabilitations des infrastructures^22,23^. La DPS fonctionne avec six bureaux (Appui technique, Gestion des ressources, Information et Recherche, Hygiène, Inspection-contrôle et Enseignement) sous le leadership d’une équipe cadre composée du Chef de Division et de 6 chefs de bureau^24^.

### Type et période d’étude

Il s’agit d’une étude mixte convergente entrant dans le cadre d’évaluation de l’approche contrat unique en RDC, cinq ans après son instauration (de 2014 à 2019). Les données quantitatives et qualitatives ont été collectées concomitamment et analysées séparément^26,27^. Les données quantitatives ont fourni des résultats préliminaires sur l’utilisation des ressources des DPS ayant opté pour l’approche CU tandis que les données qualitatives ont renseigné sur : (1) les étapes de la mise en œuvre du processus de CU et (2) la perception par les acteurs-clés des DPS, de l’impact qu’aurait eu la mise en œuvre de cette approche sur les composantes du système de santé (essentiellement les composantes ressources financières et humaines).

### Collecte des données quantitatives

Pour la collecte des données quantitatives, nous avons eu recours à la revue des documents techniques et des rapports d’activités relatives au CU des différentes directions du MSP ou des PTF. Pour les variables quantitatives, nous avons enregistré les coûts opérationnels de chacune des DPS concernées en considérant les principales lignes budgétaires retenues lors de l’Atelier de Matadi sur l’harmonisation des lignes budgétaires des DPS.

Nous avons également recueilli des données démographiques (population totale) et géographiques (superficie) de chaque DPS au cours de la même revue documentaire.

### Collecte des données qualitatives

Deux types de données qualitatives ont été recueillies : (1) les données sur la mise en œuvre du processus de contrat unique ont été recueillies par une analyse documentaire utilisant les rapports de la direction d’études et de planification (DEP) sur le CU et certains documents de référence du MSP de la RDC ; (2) des interviews individuelles semi-structurées grâce à un guide d’entretien ont été réalisés pour ressortir les opinions des acteurs-clés des DPS. Ces acteurs-clés (trois chefs de divisions, deux chefs de bureau et deux analystes au sein des bureaux des DPS) ont été sélectionnés dans les trois DPS où l’approche contrat unique a été mise en œuvre (Sud-Kivu, Nord-Kivu et Sud-Ubangi). Ils ont été interviewés à travers un appel téléphonique. L’entretien durait entre 30 et 45 minutes. Les questions visaient à comprendre l’impact du contrat unique sur les différents piliers du système de santé en province. Un cadre thématique a été conçu à l’avance en fonction des piliers qui auraient pu être intéressés par l’approche CU :

- Le financement de la santé et la gouvernance : traduisant la manière dont les DPS ayant mis en œuvre l’approche CU ont géré les ressources financières (propres ou reçues des PTF) ;
- La gestion des ressources humaines : traduisant la manière dont ces DPS ont géré le personnel tout au long de cette approche ;
- La prestation des services : traduisant l’incitation à la performance du personnel des DPS sous le régime contrat unique

### Analyses des données

Les données provenant des deux sources ont été analysées séparément puis fusionnées grâce à l’approche « side-by-side comparison » ou « comparaison côte à côte »^26,28^ : les résultats provenant des données quantitatives ont été présentés en premier, les résultats intéressants provenant des données qualitatives sont présentés en deuxième lieu. Ces deux types de résultats sont enfin fusionnés dans la discussion afin d’établir leur complémentarité par rapport à notre question de recherche.

Les données quantitatives ont été encodées dans un fichier Microsoft Excel 2016. L’analyse a été réalisée grâce au logiciel IBM Statistical Package for Social Sciences (SPSS) 24. Pour les données relatives aux coûts, nous avons calculé les coûts médians avec coûts minimum et maximum. Pour l’analyse uni-variée, nous avons calculé les médianes et leurs valeurs extrêmes (minimum et maximum) pour les variables quantitatives.

Pour l’analyse des données qualitatives provenant des interviews, nous avons effectué une analyse thématique en nous référant au cadre thématique préétabli.

L’analyse commençait par une transcription mot à mot des propos des acteurs clés, recueillis par appel téléphonique, sur un fichier Word. Ensuite, les transcriptions ont été analysés manuellement en établissant des codes qui ont été groupés ensuite en catégories. Ces catégories ont été enfin placées dans les différents groupes thématiques de notre cadre de référence. Les verbatim découlant des propos de interviewés ont être codifiés avec trois identifiants séparés par des virgules : un code comprenant leur DPS d’appartenance (en lettres, p.ex. : NK) et leur rang lors de l’appel téléphonique (en chiffre, p.ex. : 001) ; le poste occupé au sein de la DPS (en lettres, p.ex. :A), et leur sexe (M=masculin, F=Féminin).

### Considérations éthiques

L’étude a été approuvée par le comité éthique de l’Université Catholique de Bukavu (UCB/CIES/BR/014/19) et a reçu l’aval du ministère national de la santé publique en collaboration avec toutes les division provinciales de la santé de la RDC. La confidentialité a été respectée lors de la récolte des données et la présentation des résultats.

## Résultats

### Les étapes de mise en œuvre du processus

**FIGURE 1:**
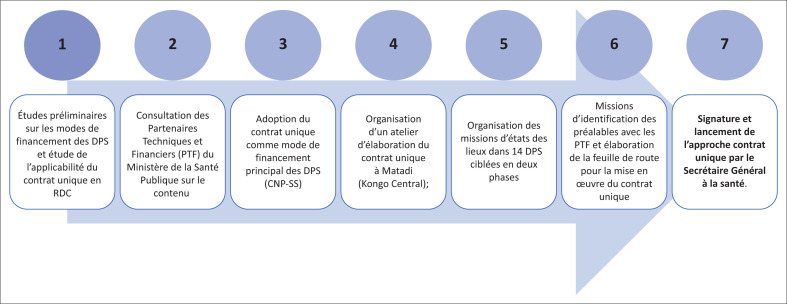
Processus de mise en œuvre de l’approche contrat unique dans les DPS.

Les étapes de la mise en œuvre de l’approche CU dans les DPS sont résumées dans la Figure 1. Le processus a nécessité 7 étapes tel que repris dans le rapport de la DEP-Santé^29^ :

- Études préliminaires sur les modes de financement des DPS et étude sur l’applicabilité du CU dans le contexte de la RDC. Cette étape a été réalisée par une équipe restreinte du MSP qui s’est appuyée sur les inégalités observées dans le financement des DPS en RDC
- Consultation des PTF sur le contenu et le processus de mise en œuvre du CU en RDC (24 /11 au 22/ 2014). Cette étape a permis au MSP d’obtenir l’alignement des différents PTF dans la mise en œuvre de l’approche CU. Mais deux observations ont été faites par les PTF :
  ■ Les PTF ont insisté sur la participation financière progressive du gouvernement dans le temps de manière à couvrir, à terme, au moins 70% du budget des DPS;
  ■ Certains PTF ne maitrisent pas des contraintes liées aux bailleurs des fonds (disponibilité, modalité et rythme de versement des fonds) car les décideurs sont au niveau des sièges en particulier pour les agences internationales.
- Adoption officielle du CU comme mode de financement principal des DPS en RDC par le CNP-SS en décembre 2014.
- Définition des activités de base et des coûts opérationnels des DPS à considérer dans le CU:
  ■ les experts du MSP et les PTF ont défini, en février 2015, la liste minimale des activités essentielles de la DPS (paquet d’activités de base de la DPS), qui devraient être exécutées pour garantir le bon fonctionnement du système de santé en province. Il s’agit : (1) du fonctionnement minimal de la DPS qui définit l’existence même de cette institution (équipe minimum et subsides pour le fonctionnement validé par le CNP-SS), (2) de l’encadrement des ZS (une mission de 7 jours par trimestre dans chaque ZS) et (3) des activités de pilotage et de coordination du système de santé en province. Les coûts de fonctionnement des DPS ont été répartis en six lignes budgétaires, soit une ligne spécifique pour chacune des deux premières activités de base alors que la troisième activité a été éclatée en quatre lignes pour besoin de clarté. Pour faciliter la compréhension, nous avons présenté les coûts des DPS de manière globale avec des spécificités par ZS de santé soit par habitant dans le Tableau 1.
- Organisation des missions d’états des lieux dans les provinces ciblées pour évaluer les coûts réels applicables à l’approche CU et les ressources disponibles.
- Mission conjointe MSP et PTF pour l’identification des préalables à la signature du CU et élaboration de la feuille de route pour sa mise en œuvre
- Signature et lancement de l’approche CU par le Secrétaire Général à la santé (la première DPS a signé le contrat unique en juillet 2016, soit près de 2 ans après le lancement du processus).

**TABLEAU 1:** Coûts opérationnels des DPS en 2017 en RDC.

Ligne budgétaire ou rubrique	Médiane	Min	Max
Nombre des zones de santé	21	12	36
Nombre du personnel	55	40	75
Subsides Fonctionnement (USD)	$ 481 818.00	$ 353 875.00	$ 599 474.00
Coût des missions Encadrement ZS et suivi du PAO (USD)	$ 137 148.00	$ 93 700.00	$ 339 894.00
Coût des activités de pilotage et coordination système de santé (USD)	$ 145 538.00	$ 93 699.00	$ 296 743.00
Total budget de la DPS (USD)	$ 789 948.50	$ 575 657.00	$ 1 161 961.00
Population	3 542 529	1 924 259	9 119 434
Superficie (km^2^)	94 717	9 956	197 637
Densité (hab./km^2^)	43	15	916.0
Contribution complémentaire du budget de l’État (% budget global)	21.0	9.0	34.0

USD, United States dollar; PAO, Plan d’action opérationnel; hab., habitant.

### Profil et coûts opérationnels d’une DPS standard en RDC (synthèse données 2017)

La contribution du Gouvernement en complément au budget global des DPS varie entre 9 et 34% selon les provinces dont 80% – 90% étaient consacrés principalement aux primes du personnel. Cette proportion est donc plus élevée dans les provinces où il y a une grande proportion d’agents pris en charge par l’État (salaires et primes diverses).

Le seuil de 43 hab./km^2^ a été considéré pour catégoriser les DPS à faible et à forte densité démographique.

**FIGURE 2:**
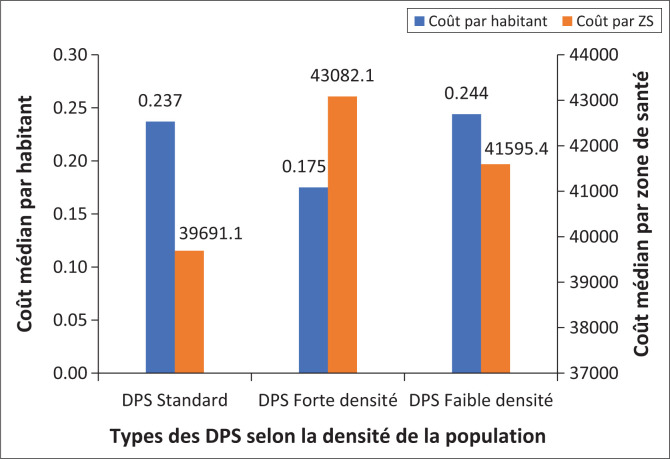
Coûts opérationnels des DPS en RDC en fonction de la densité de la population, 2017

Nous avons identifié deux indicateurs susceptibles d’aider à estimer le coût d’une DPS en RDC : soit le coût médian par habitant, soit le coût médian par ZS :

- Les coûts les plus élevés sont observés dans les provinces ayant une population dispersée et avec des problèmes d’accessibilité géographique. En considérant le seuil d’une densité médiane de 45 habitant (hab)/km^2^ les provinces à faible densité ont un coût plus élevé (coût par habitant 0.244$ et coût par ZS de 41594.4$) alors que celles avec une densité supérieure à 45 hab/km^2^ ont un coût relativement faible (coût par habitant de 0.175$ et coût par ZS de 43 082.1 $). Les détails des coûts sont présentés dans le la Figure 2.
- Les subsides de fonctionnement de la DPS prennent près de 60% du budget global. La contribution du Gouvernement au budget global variait entre 9 et 34% selon les provinces dont 80% – 90% était consacrée principalement aux primes du personnel

### Niveau de progrès réalisé dans la mise en œuvre du CU dans les DPS

**FIGURE 3:**
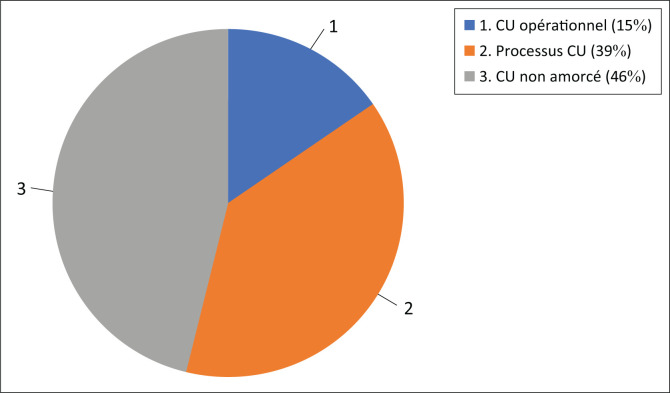
État de mise en œuvre du CU dans les DPS, 2017. Niveau mise en oeuvre contrat unique début 2017 (*n* = 26 DPS).

Au 31 décembre 2016 soit deux ans après le lancement du processus, 14 DPS sur 26 (soit 53,8%) avaient réussi à mobiliser le financement pour démarrer le processus de mise en œuvre du CU (Figure 3) : Bas-Uélé, Haut-Katanga, Haut-Lomami, Haut-Uélé, Kwango, Kwilu, Kongo-Central, Lualaba, Mai-Ndombe, Maniema, Nord-Kivu, Sud-Kivu, Sud-Ubangi et Tshopo. Toutefois, seulement 4 DPS avaient accompli toutes les étapes préparatoires et donc ont commencé la mise en œuvre du contrat unique en 2017. Il s’agit des DPS du Nord Kivu, Sud Kivu, Sud Ubangi et Tshopo.

### Perception de l’impact, par les acteurs-clés, de la mise en œuvre du CU sur les composantes du système de santé dans les DPS

Après analyse des données provenant des opinions des différents acteurs des DPS (Nord Kivu, Sud Kivu, Sud Ubangi) ayant mis en œuvre le contrat unique, trois thèmes ont émergé en se basant sur les composantes d’un système de santé :

#### Financement de la santé et Gouvernance

Concernant le financement de la santé, certains aspects ont été soulevés par les interviewés quant à l’impact du contrat unique sur le vécu financier de la DPS. Il s’agissait principalement de :

**La réduction de la fragmentation du financement des DPS:** Le contrat unique semble avoir apporté une certaine solution au problème de planification lors de l’utilisation des ressources pour certains acteurs de la DPS.

« … Un peu d’avancée sur la visibilité/lisibilité des financements. Les planifications sont concertées et parfois les gaps à combler sont connus de tous. » (NK002, B, M)

Mais, la plupart des participants n’étaient pas d’accord avec l’idée que le CU aurait permis une réduction réelle de la fragmentation du financement de leur DPS. Les financements des programmes spécialisés sont encore gérés par l’autorité nationale (programmation verticale) et les partenaires ne se sont pas encore alignés à la logique d’un panier commun de financement :

« La DPS n’a joué que le rôle de fudiciaire pour les programmes spécialisés. Le financement des programmes spécialisés demeure vertical et fléché aux activités spécifiques. » (SU001, A, M)

Par ailleurs, certains partenaires n’acceptent pas que leurs financements soient utilisés pour d’autres programmes qui ne sont pas leur vision ; et donc le CU ne semble pas apporter quelque chose de nouveau au mode de financement de la DPS. Aussi, certains partenaires continuent à utiliser leurs fonds sans pour autant le déverser dans le basket fund :

« Le fait du principe de caisse virtuelle fait que le mode de financement avant et après le CU reste le même. Et aussi la quasi-totalité des partenaires quoique ayant souscrit au CU continuent de venir avec des protocoles d’accord à signer avec la DPS. » (SK003, C, M)

« … car les financements de certains programmes restent encore verticaux et spécifiques notamment ceux financés par le Fond Mondial et GAVI. Par ailleurs plusieurs autres financements ne peuvent être utilisés que pour les lignes auxquelles ils ont été destinés, bien que les comptes utilisés soient ceux de la DPS, cette dernière sert tout simplement de transit sans flexibilité. D’autres partenaires continuent à gérer leur financement, c’est le cas de PROSANI USAID. » (SK002, B, M)

« … parce que les financements des programmes spécialisés bien que passant par les comptes de la DPS ne sont destinés qu’aux activités des programmes selon les directives venant des directions des programmes. » (SU001, A, M)

**Renforcement du partenariat et de la coordination des financements:** Le partenariat entre la DPS et les PTF semble bien fonctionner avec l’avènement de l’approche contrat unique. Néanmoins, certains partenaires restent figés aux programmes verticaux des PTF en abandonnant les autres programmes qui ont besoin d’un financement pour fonctionner.

« Oui … le processus du partage d’information sur les activités à mener ainsi que les budgets y afférents, est déjà effectif en dépit du fait que plusieurs partenaires ne sont pas encore alignés. » (NK001, A, M)

« … Oui, le contrat unique a renforcé le partenariat qui existait avec la DPS mais n’a pas amélioré la coordination des financements. » (SK002, B, M)

Certains partenaires ne semblent pas se rallier à l’idée de partager avec la DPS leurs plans d’activités et budgétaires détaillés :

« … mais partiellement car la persistance des protocoles spécifiques met à mal cette coordination. Certains partenaires ayant souscrit au CU ne partagent pas les informations financières détaillées quant à leur appui à la réalisation des missions de la DPS. » (SK003, C, M)

L’approche contrat unique aurait renforcé la redevabilité de la DPS envers ses partenaires ainsi que le sens de responsabilité :

« La responsabilisation, la redevabilité qui se sont améliorées car les planifications et les évaluations sont conjointes. » (SK002, B, M)

« Le CU a renforcé la redevabilité de la DPS vis-à-vis de ses partenaires et du Gouvernement. » (SK003, C, M)

**Gestion des ressources financières de la DPS:** Le contrat unique a aidé à améliorer la gestion des ressources provenant des partenaires financiers de la DPS. Avec les procédures de gestion qui sont partagées entre les différentes parties prenantes et les évaluations internes qui se font régulièrement, cette approche semble apporter un « plus » au système de gestion des ressources :

« … avec les évaluations périodiques du CU, les rapports financiers de la DPS sont partagés systématiquement avec l’ensemble des parties prenantes … » (SK003, C, M)

« … la transparence dans la gestion a été effective, le CU a permis la lisibilité de financement de chaque intervenant dans la DPS… un logiciel de gestion a été mis en place pour suivre le financement des partenaires. » (SU001, A, M)

« En effet, leur utilisation s’appuie sur des procédures claires. Par ailleurs, au terme des audits financiers dont la DPS est bénéficiaire, les recommandations d’amélioration formulées ne touchent pas aux fondamentaux en matière de gestion [*Outils disponibles et à jour et pas de tricherie*]. » (NK001, A, M)

« … Le contrat unique a apporté une lisibilité dans le financement des activités au niveau de la DPS réduisant ainsi le double financement, la transparence dans la gestion a été effective … » (SU001, A, M)

#### Gestion des ressources humaines

**Maîtrise du personnel et description de poste:** Pour certains, le contrat unique a permis de renforcer la DPS dans la définition de la description de poste pour tout agent qui serait recruté, hormis les quelques cas d’influence de la politique :

« … la lisibilité des ressources signalées dans le CU oriente toute décision en rapport avec le recrutement. Par ailleurs, tout recrutement se réfère au cadre et aux structures organiques et livre des emplois du ministère de la santé ; et passe par un appel à candidature. Ces outils précisent les descriptions de poste de cadres et d’agents de la DPS… » (NK001, A, M)

« Avec la réforme du système de santé au niveau intermédiaire, le personnel qui doit prester à la DPS a été défini dans le cadre organique du ministère de la santé et le livre des emplois. Les recrutements se font selon les besoins présentés par les bureaux. » (NK002, B, M)

D’autres, par ailleurs, pensent que la description des tâches existait déjà avant l’avènement du contrat unique et que la rémunération ne suit pas jusque-là. Aussi, les textes sont très globaux et ne permettent pas d’établir une description de poste claire :

« Une description de poste a été fait pour chaque Agent de la DPS indépendamment du Contrat unique. Par contre, la motivation n’a pas suivi dans le cadre de contrat unique. » (SU001, A, M)

« Non pas du tout. Le livre des emplois reste globalisant sans description claire des descriptions de poste pour chaque agent. » (SK003, C, M)

**Rémunération du personnel:** L’approche contrat unique n’a pas permis d’améliorer la rémunération des membres de la DPS qui vivent, pour la plupart des primes de l’État. Par ailleurs, la prime des PTF qui adhère au renforcement de la rémunération du personnel de la DPS ne concerne que certaines personnes. La cause la plus incriminée est le manque d’adhésion des partenaires à l’approche contrat unique. Cette situation aurait occasionné le départ de certains agents de la DPS :

« … la grille barémique convenue entre le GIBS et Ministère, comme référence dans la motivation du personnel de la DPS dans le cadre de contrat unique, n’a jamais été utilisée. De ce fait, il n’y a pas eu la complémentarité recherchée dans le contrat unique dans le volet de la motivation. En revanche, certains partenaires alignés dans le payement de complément de rémunération à la DPS se sont retirés au nom du contrat unique. Le contrat unique a eu sur ce point des effets pervers. » (SU001, A, M)

« … le barème retenu lors du costing n’est pas encore appliqué. En effet, un seul partenaire soutient les primes du personnel. » (NK001, A, M)

« … seul deux partenaires payaient des primes à une certaine catégorie du personnel en plus des primes du gouvernement … » (SK003, C, M)

**«** Peu de partenaires interviennent dans la rémunération du personnel … le niveau actuel des rémunérations (salaires et primes) reste donc peu satisfaisant surtout pour les cadres et les personnels sans primes de risque ni salaires … des cas observés de fuite des cerveaux hors de DPS. » (NK002, B, M)

#### Prestation des services (incitation à la performance)

Il a été remarqué une amélioration de l’incitation à la performance des DPS avec l’avènement de l’approche contrat unique.

« … le CU met en place un cadre de performances et recommande aux parties prenantes de signaler les ressources nécessaires pour la mise en œuvre des activités. Ainsi, ces outils guident les actions à mener et contribuent à leur rationalisation. » (NK001, A, M)

Par contre, certains acteurs estiment que cette approche n’a pas eu d’influence majeure sur la performance de la DPS et qu’elle a apporté une démotivation probablement liée à la disparité dans la rémunération des agents. D’autres pensent qu’il faudra renforcer certains aspects pour améliorer la performance des DPS :

« Non, je ne saurai attribuer la performance de ma DPS au contrat unique qui par contre a amené une démotivation qui a impacté, dans l’évolution de sa mise en œuvre, sur cette performance. » (SU001, A, M)

« … les évaluations restent peu constructives : limitation au cadre de performance DPS, évaluateurs peu compétents ou trop changeants, le cadre de performance nécessitant des adaptations … » (NK002, B, M)

## Discussion

### Des coûts des DPS

La RDC est un pays vaste composée de 26 provinces avec une grande diversité par rapport à la répartition démographique et à la praticabilité des réseaux de communications inter-provinces et intra-province. Certaines provinces étant servies plus par des réseaux routiers dont plus de 90% sont des routes sont moins praticables; d’autres par des réseaux maritimes dont la praticabilité dépend du sens de navigation du fleuve en général ; et au sein des provinces certaines ZS ne sont accessibles que par voie aérienne^16,22^. Cette situation ne permet pas au niveau central d’envisager une planification standardisée des activités devant se dérouler en provinces et dans les ZS. Le travail réalisé dans le cadre de cette étude a permis de mettre en évidence certains facteurs susceptibles de soutenir le processus de planification au niveau intermédiaire dans le contexte de la RDC. Cette étude a montré que les coûts des DPS peuvent être estimés soit sur base de la densité qui intègre à la fois la population et la superficie ; soit sur base du nombre des ZS couvertes par la DPS. Ces coûts varient autour de 0.24 USD par habitant et 40 000.00 USD par ZS. Des interventions pilotes sont nécessaires pour confirmer l’applicabilité de cette approche dans la planification à long termes et la dynamique observée dans la variation des coûts avec l’accroissement démographique. Néanmoins ceci ne doit pas étouffer les recommandations de la SRSS qui prône la décentralisation des centres de négociation des financements au niveau provincial^13^.

### Effets du contrat unique sur le renforcement du système de santé (DPS)

#### Effet sur la gestion des ressources financières

La plupart des acteurs de différents DPS sont d’accord sur le fait que l’approche contrat unique ait apporté des éléments de plus à la manière de gérer les ressources financières (création d’un cadre de concertation réunissant à la fois les PTF et autorités du MSP à la DPS ; suivi transparent des financements à travers un logiciel ; système d’audits renforçant la redevabilité). Ces piliers de gestion de ressources financières vont dans le sens de la déclaration de Paris^30^ qui préconise que le financement extérieur puisse s’aligner dans la vision et les objectifs sanitaires du système de santé appuyé.

Néanmoins, certains partenaires semblent garder une approche verticale de leurs financements ne permettant pas que les autres programmes bénéficient de ce qu’ils offrent à la DPS.^31^ Ceci pourrait altérer encore l’un des principes fondamentaux de cette approche qui est la réduction de la fragmentation des ressources de la DPS. Les financements des PTF proviennent souvent des bailleurs de fonds ayant un objectif bien précis et qui n’autorisent pas souvent que leurs fonds soient alloués à d’autres activités, quel que soit le niveau de priorité^32,33^. C’est à ce niveau que la DPS et l’IPS devraient encore faire des plaidoyers pour que les PTF puissent réellement déverser leurs financements dans un panier commun, puisque déjà le système de suivi semble marcher correctement.

#### Effet sur la gestion des ressources humaines et l’incitation à la performance

Le basket found a permis, selon certains acteurs des DPS, de renforcer la maitrise du personnel avec une description des tâches assez précise. Ceci pourrait être lié au fait que l’approche contrat unique se retrouve dans une grande réforme du système de santé qui a été instaurée en RDC en 2014^32,33^. Cette réforme a abouti à la création de la DPS ayant plusieurs bureaux de coordination des programmes bien définis au niveau intermédiaire (au niveau de la province).

Mais, bien que le fonctionnement de base de cette approche prévoit des primes minimums pour tous les agents^14^ , certains acteurs pensent que cela n’est pas encore effectif car tous les PTF n’interviennent pas dans cette rémunération. Et donc, l’on est pas encore strictement sorti dans le système de gestion par projet qui ne pouvait rémunérer que quelques agents en fonction de leurs rôles respectifs dans différents projets^14^. Il faudra donc améliorer cet aspect afin de garantir un climat de satisfaction et inciter ainsi la performance des agents de la DPS.

Le gap de la rémunération des agents devrait être comblé par le financement du gouvernement congolais. Aussi, la part des PTF dans les primes octroyées au personnel des DPS reste élevée, ce qui dénoterait d’une large dépendance au financement extérieur, d’autant plus que l’engagement du pays pour financer le système de santé reste encore en dessous de 15% de son budget annuel tel que préconisé par l’accord d’Abuja^34^. Du coup, les PTF semblent occuper une place assez importante dans le financement de la santé et donc dans la rémunération des agents de la DPS. parce que même pour arriver au 15%, les pays africains comptaient sur des donateurs extérieurs. Le contrat unique, appliqué tel que préconisé, constituerait ainsi un élément capital dans la motivation à la performance des agents des DPS.

### Des défis majeurs à la mise en œuvre de l’approche contrat unique

L’analyse du travail à mi-parcours de la mise en œuvre du contrat unique dans les quatre DPS de la RDC montre qu’il a encore des défis à surmonter pour stabiliser et renforcer l’approche contrat unique :

- La persistance d’un décalage entre la planification budgétaire de certains PTF et le plan de travail trimestriel des DPS. Ceci est à la base des irrégularités et de l’asynchronisme dans le financement des DPS.
- Pour certains PTF, les responsables locaux n’ont pas de marge de manœuvre suffisante pour la prise des décisions par rapport aux procédures de décaissement et de justification des fonds ainsi que l’organisation d’un audit en commun.
- Tous les PTF ne se sont pas encore alignés sur l’approche contrat unique et continuent à financer les activités (Bureaux de la DPS et Programmes) de manière isolée.
- La non effectivité de la progressivité de la part du financement de Gouvernement (national et provincial) au fonctionnement des DPS.

Ces défis méritent être relevés avant toutes décision d’extension du CU dans d’autres provinces.

### Limites de l’étude

Notre étude présente certaines limites. Il s’agit essentiellement des résultats qui ont décrit le processus au lieu d’aller dans les résultats de la mise en œuvre de cette approche contrat unique. Cela peut être justifié par le fait que cette étude est considérée comme plutôt normative, décrivant le processus et quelques résultats globaux intermédiaires et que l’évaluation finale, s’inspirant de ces résultats, pourrait mieux ressortir ces éléments manquants. Aussi, le processus n’est déjà effectif que dans certaines DPS, et donc nous nous sommes basé sur l’expérience de ces DPS considérés comme « pilotes » pour ressortir les leçons à tirer de la mise en œuvre de l’approche contrat unique en RDC.

## Conclusion et leçons tirées

Les résultats de notre étude montrent que même si la mise en œuvre des réformes du financement de la santé dans des « États fragiles » est laborieuse et complexe, la mise en place d’un cadre de concertation des partenaires techniques et financiers peut faciliter la décentralisation effective du processus de planification et la mise en place d’un basket-fund virtuel au niveau des provinces décentralisées. Cette stratégie est plus efficiente car elle garantit la participation effective des entités décentralisées au processus de négociation des financements et permet d’intégrer les spécificités des entités décentralisées dans la planification et la gestion des financements du secteur santé dans des « États fragiles » aux dimensions géographiques larges comme la R.D.C. Les actions futures de redressement de cette approche doivent se focaliser sur les plaidoyers visant à aligner tous les PTF sur les plans d’action opérationnels des DPS et l’augmentation des subsides de l’état congolais pour accompagner ces plans des DPS.

## Remerciements

Nous remercions la DEP (Direction d’étude et Planification) pour son appui technique lors du déroulement de ce travail.

### Intérêts concurrents

Les auteurs déclarent qu’il n’existe aucun conflit d’intérêt.

### Contributions des auteurs

G.B. a contribué de façon substantielle à la conception de l’étude, la collecte des données, l’analyse des données et la discussion des résultats et a contribué significativement à l’écriture et la révision du manuscrit. S.M. a contribué de façon substantielle à la conception de l’étude, la collecte des données, l’analyse des données et la discussion des résultats et a contribué significativement à l’écriture et la révision du manuscrit. H.K. a contribué de façon substantielle à la discussion des résultats et a contribué à la révision du manuscrit. C.M. a contribué de façon substantielle à la discussion des résultats et a contribué à la révision du manuscrit. R.N. a contribué de façon substantielle à la collecte des données et à la révision du manuscrit. A.Y. a contribué de façon substantielle à la collecte des données et à la révision du manuscrit. F.C. a contribué de façon substantielle à la conception de l’étude, la collecte des données, a contribué significativement à la discussion des résultats ainsi qu’à l’écriture et la révision du manuscrit.

### Information sur le financement

Ce travail a été réalisé grâce au financement de RIPSEC-RDC (Renforcement Institutionnel des politiques de Santé basées sur les évidences en R.D. Congo).

### Déclaration des disponibilités de données

Les données dont découlent les résultats de cette étude sont disponibles via l’auteur correspondant (SM) sur demande raisonnable.

### Avis de non responsabilité

Les opinions exprimées dans cet article proviennent des données récoltées et analysées par les auteurs et ne sont position officielle d’aucune institution.
